# Mirror-centered representation of a focusing hyperbolic mirror for X-ray beamlines

**DOI:** 10.1107/S1600577524009603

**Published:** 2024-10-29

**Authors:** Jean-Pierre Torras

**Affiliations:** a Linac Coherent Light Source SLAC National Accelerator Laboratory 2575 Sand Hill Road Menlo Park CA94025 USA; Tohoku University, Japan

**Keywords:** X-ray optics, mirror-centered hyperbolic shape, hyperbolic mirrors, focusing optics, mirror bending, Wolter type configurations

## Abstract

Conic sections are commonly used in reflective X-ray optics. Hyperbolic mirrors can focus a converging light source and are frequently paired with elliptical or parabolic mirrors in Wolter type configurations. This paper derives the closed-form expression for a mirror-centered hyperbolic shape, with zero-slope at the origin. Combined with the slope and curvature, such an expression facilitates metrology, manufacturing and mirror-bending calculations. Previous works consider ellipses, parabolas, magnifying hyperbolas or employ lengthy approximations. Here, the exact shape function is given in terms of the mirror incidence angle and the source and image distances.

## Abstract 2

The derivation of the closed-form expression for a focusing hyperbolic mirror shape in a mirror-centered arrangement ideal for metrology, manufacturing and mirror bending is presented.

## 1. Introduction

Shaped mirrors are used in reflective optics for various purposes. X-ray optics frequently employ conic sections such as ellipses, parabolas and hyperbolas. An elliptical mirror provides point-to-point beam focusing, a parabolic mirror can focus a collimated beam or collimate a diverging beam, and a hyperbolic mirror can be used in four different configurations. Generally, a hyperbolic mirror can focus a converging light source or magnify a diverging light source. More specifically, a hyperbolic mirror can focus a real image with a virtual source or defocus (extend or magnify) a beam by generating a virtual image with a real source. In each case, the mirror–image distance can be greater or less than the source–mirror distance, therefore totaling four distinct mirror configurations.

Wolter type (Wolter, 1952 ▸) optical systems typically use hyperbolic mirrors in combination with a parabolic or elliptical mirror. Lider (2019 ▸) provides an extensive overview of Wolter and other common focusing optics. A Wolter type setup has numerous advantages including reduced aberrations and improved focusing stability (Pareschi *et al.*, 2021 ▸). In X-ray beamlines, hyperbolic mirrors are frequently seen in Wolter-like configurations such as for advanced Kirkpatrick–Baez optics (Yamada *et al.*, 2019 ▸) or zoom condensers (Matsuyama *et al.*, 2021 ▸). Though less common in everyday applications, hyperbolic mirrors can be also be found in astronomical optics such as the classic Cassegrain telescope (Schroeder, 2000 ▸).

Mathematical descriptions of Wolter optics and mirror surface profiles in various layouts have been extensively explored. VanSpeybroeck & Chase (1972 ▸) provide one of the earliest investigations into Wolter type I mirror surface equations in terms of the source and image distances and incidence angle. Saha (1987 ▸) went on to illustrate numerous other Wolter configurations useful for telescope design.

Mirror-centered ellipse expressions are numerous; Padmore *et al.* (1996 ▸) was perhaps the first to derive one as a power series approximation. Later investigations by McKinney *et al.* (2011 ▸) provide exact expressions for ellipses and parabolas. However, expressions for hyperbolas are difficult to find, typically encountered only as cursory references (Rah *et al.*, 1997 ▸).

More recently, works on exact mirror-centered expressions for ellipses (Goldberg, 2022*b* ▸), parabolas (Goldberg, 2022*a* ▸) and magnifying hyperbolas (Goldberg & Sanchez del Rio, 2023 ▸) have been published. The derivation method herein relies on similar techniques described by Goldberg & Sanchez del Rio (2023 ▸).

This report derives a new, closed-form expression for a focusing plane–hyperbolic mirror surface. The derivation characterizes a hyperbolic mirror in terms of the optical source and image distances and incident grazing angle. The expression describes a mirror-centered arrangement, with zero slope and the mirror center at the origin. Furthermore, this report derives expressions for the slope and curvature. Describing the mirror in this form minimizes the slope along the shape. These expressions are most convenient for metrology, mirror manufacturing (Shi *et al.*, 2016 ▸) and mirror-bending calculations. The curvature equation can easily be used to determine the bending moments necessary to generate a hyperbolic surface profile on a roughly beam-shaped mirror (Howells *et al.*, 2000 ▸). Such bending allows for dynamic focusing and correction of surface-shape errors (Zhang *et al.*, 1998 ▸).

## 2. X-ray mirror parameters

The standard method for specifying a mirror shape uses the source distance *p*, image distance *q* and grazing angle θ. In X-ray beamlines, we typically use focal lengths on the order of meters, while mirrors typically have lengths from tens of millimetres up to 1.5 m. The incidence angle θ needed for total external reflection of an X-ray beam depends on the mirror material and coating. For X-rays, θ is usually on the order of milliradians. Here, the incidence angle is grazing, measured from the mirror surface. Cited works often use an incidence angle measured from the normal to the mirror surface. Additionally, it is common to define the source and image distances as positive scalars. We may be tempted to define the virtual image of a defocusing hyperbolic mirror negatively because it lies upstream of the mirror. However, for hyperbolic mirrors, we define *p*, *q* > 0 and *p* ≠ *q*.

Each institution has its own coordinate system for describing the beam direction and the mirror surface. In this paper, we only consider the planar mirror case in the *xy* plane. The beam moves roughly in the −*x* to +*x* direction. The *x* coordinate describes the position along the mirror surface (tangential mirror direction), and the mirror surface height (or shape profile) is a function *y*(*x*). There is no variation in the sagittal mirror (*z*) direction since we only consider a plane–hyperbolic mirror.

We use an east–west-opening hyperbola in this coordinate system. The west (or left) facing hyperbola represents a focusing hyperbolic mirror, whereas the east (or right) facing hyperbola represents a defocusing hyperbolic mirror. After performing the necessary transformations, we find that the foci of a focusing hyperbola lie in quadrants I and IV (downstream of the mirror), whereas the foci of a defocusing hyperbola lie in quadrants II and III (upstream of the mirror).

When we characterize the concavity of an optical mirror (convex or concave), we apply the polygon concavity definition (Wikipedia, 2024*a* ▸) rather than the concavity of a mathematical function (Wikipedia, 2024*b* ▸). A convex mirror has d^2^*y*/d*x*^2^ < 0, whereas a concave mirror has d^2^*y*/d*x*^2^ > 0.

The mirror radius of curvature, a common figure of merit, is often approximated as the inverse of the second-order derivative, [1/(d^2^*y*/d*x*^2^)]. The exact radius of curvature, *R*, in fact depends on the slope as well, 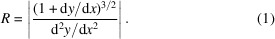
The ramifications of the approximation become evident in mirror-bending theory, as explored by Mao *et al.* (2011 ▸).

## 3. Analytical solution

**Figure 1:**
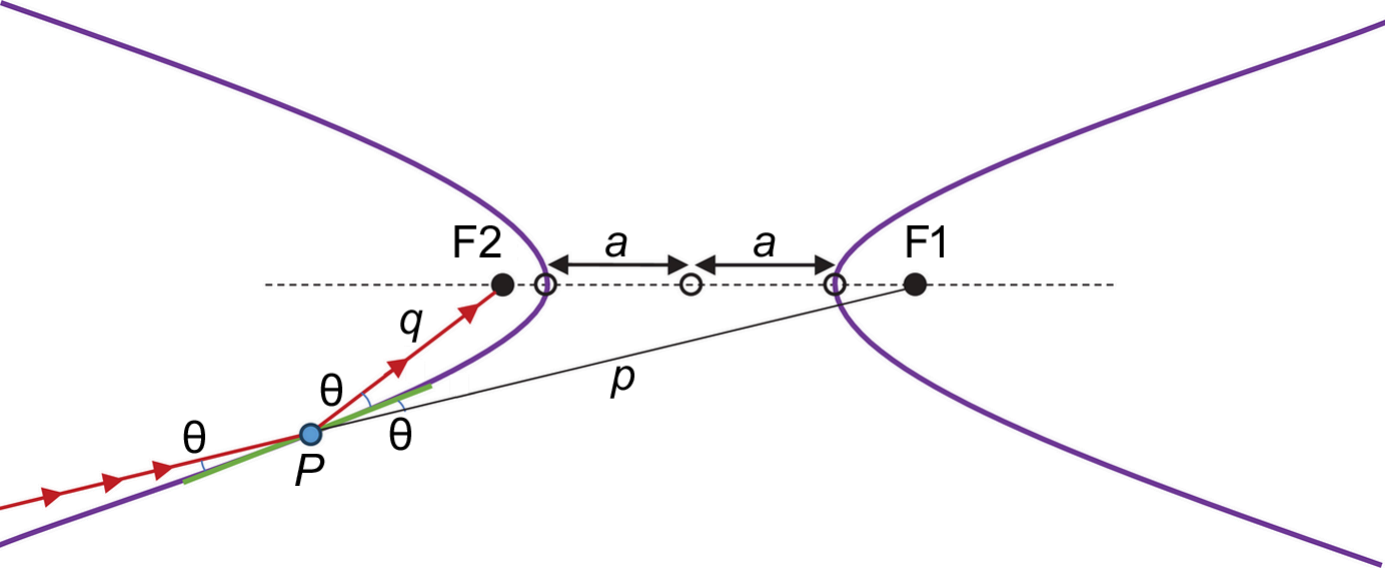
Concave focusing hyperbola.

We can construct a hyperbola from a set of points whose difference of distances from two fixed foci is a constant value (Wikipedia, 2024*c* ▸). In other words, for any arbitrary point, *P*, on the surface of a hyperbola with focal points, *F*_1_ and *F*_2_, and major axis, 2*a*, 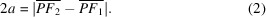
The canonical hyperbola equation depends on terms *a* and *b*, the semi-major and semi-minor axes, respectively, 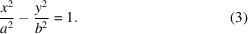
Particularly for X-ray beamlines, it is more convenient to define optics in terms of the source and image distances and incidence angle. Therefore, we let parameters *p* = *PF*_1_ and *q* = *PF*_2_ such that
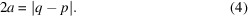
Equation (4) shows why we must have *p* ≠ *q*. A focusing hyperbolic mirror generates a virtual source with a real image in two distinct configurations. Fig. 1 ▸ shows a concave focusing hyperbola with *q* < *p*. In this configuration, the (real) mirror–image distance is *shorter* than the (virtual) source–mirror distance. Such a hyperbolic mirror is typical for a Wolter type I optical system. The unidirectional arrows depict the beam path.

**Figure 2:**
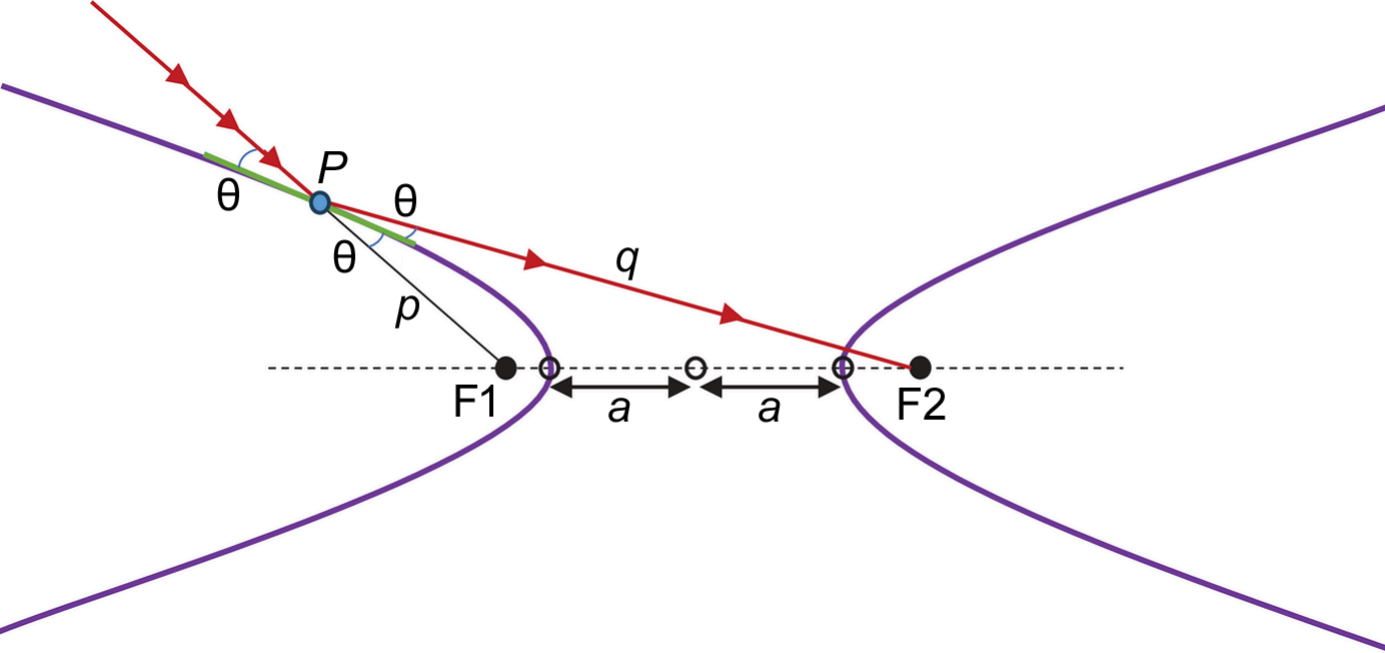
Convex focusing hyperbola.

Fig. 2 ▸ shows a convex hyperbola with *q* > *p*. In this configuration, the (real) mirror–image distance is *longer* than the (virtual) source–mirror distance. Such a hyperbolic mirror is typical for a Wolter type II optical system. Both configurations focus the beam to the hyperbola’s focal point, F2. We continue the derivation from here using the convex hyperbola.

**Figure 3:**
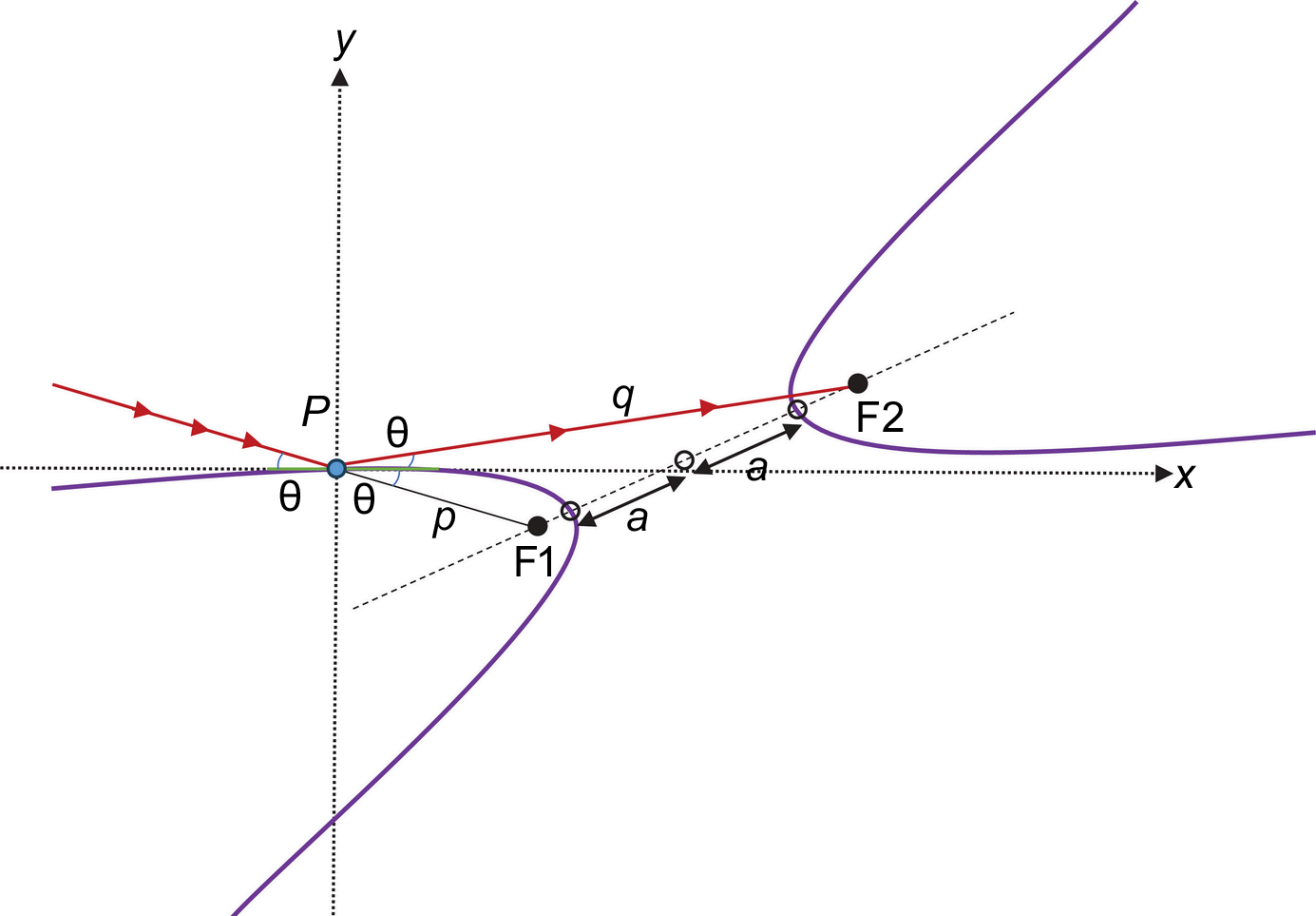
Mirror-centered convex focusing hyperbola.

Next, we rotate the hyperbola counterclockwise, and translate to the desired mirror-centered orientation with d*y*/d*x*(0) = 0 and *y*(0) = 0 (*i.e.* the mirror slope is tangent to the *x* axis at [*x*, *y*] = [0, 0]) as shown in Fig. 3 ▸. Another useful property of hyperbolas becomes evident in this orientation. The tangent to the hyperbola at the origin (which is now the tangent to the *x* axis) bisects the angle between lines *PF*_2_ and *PF*_1_, or *q* and *p*, respectively. This bisecting angle is the incidence angle θ.

We analytically determine the coordinates of the hyperbola foci relative to the mirror center at the origin as 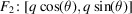 and 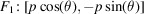. The real image is located at *F*_2_, while the virtual source originates from *F*_1_.

From equation (4) we can say
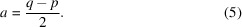
We let an arbitrary point *P* on the hyperbola have the coordinates [*x*, *y*]. We apply the distance formula for all points *P* to satisfy the hyperbola construction from equation (4),
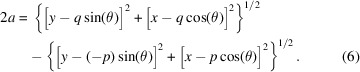
Ultimately, we want an expression of the hyperbola shape, *y*(*x*), dependent on terms [θ, *p*, *q*]. First, we separate the square roots, square both sides, isolate the remaining square root and square again (Goldberg & Sanchez del Rio, 2023 ▸). From here on, we suggest employing mathematical software: we used *Wolfram Mathematica* (Wolfram Research, 2024 ▸).

We can substitute in some intermediary terms, *m* and *n*, to facilitate this process, so we let
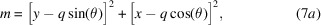

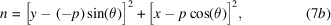
such that equation (6) becomes
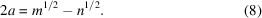
We follow the aforementioned squaring process to expand equation (8) to
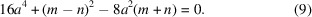
In turn, we find that equation (9) is much easier to manipulate than equation (6). Returning to our goal of solving for a function *y*(*x*), we substitute the [*y*, *x*, θ, *p*, *q*] terms from equation (7) and the [*p*, *q*] terms from equation (5) into equation (9). We expand the binomial, factor out similar *y* terms and simplify to get a quadratic equation of the form
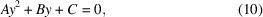
such that
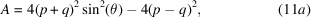

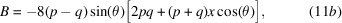

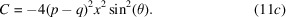
We apply the quadratic formula to *y* and simplify to obtain a raw equation for the shape profile of a plane–hyperbola *y*(*x*), dependent on terms [*p*, *q*, θ], 
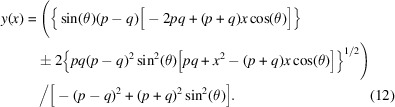
Recall Figs. 1 ▸ and 2 ▸; in this form, we must use the negative root to express the left (concave) hyperbola when *q* > *p* because there is a (*p* − *q*)^2^ term in the root. Similarly, we use the positive root to express the left (convex) hyperbola when*q* < *p*. However, we can simplify the equation to a more convenient form by reducing the terms in the root, letting*2a* = *q* − *p* from equation (5), and letting 2*k* = *q* + *p*, 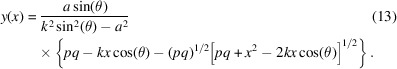
By removing the (*p* − *q*)^2^ term from the root, we have an extra term to provide a sign change depending on the relative values of *p* and *q*. Therefore, with equation (13), we only need the negative root case to define the shape profile regardless of whether *q* < *p* or *q* > *p*. Additionally, we advise keeping the root products separated to provide a red flag in case one erroneously uses *q*, *p* < 0. If we used *q*, *p* < 0 with the root products combined under a single root [or in the form of equation (12)], the resulting mirror shape would not function as intended (in fact we could get an ellipse). Next, we provide the mirror slope d*y*/d*x* and the second derivative 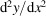. For brevity, we describe the derivatives with the following terms, 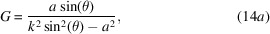

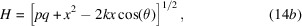

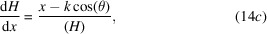

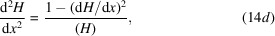
such that
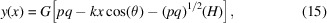

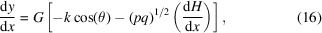

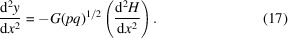
As mentioned previously, a focusing hyperbolic mirror with*p* < *q* produces 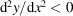 and is convex. When *p* > *q*, then 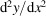 > 0 and the mirror is concave. We can also plug equations (16) and (17) into equation (1) to calculate the radius of curvature. Appendix *A*[App appa] provides the supplementary equations for a defocusing hyperbolic mirror.

## 4. Alternative solutions

Previous works have provided numerically identical or similar solutions to the equations presented.

Rah *et al.* (1997 ▸) delivered a single expression encompassing the mirror-centered shape of an ellipse, parabola and hyperbola. By choosing an appropriately negative combination of *p*, *q* values (*r*, *r*′ in the paper) and using an incidence angle measured from the normal (as opposed to a grazing angle), we can manipulate the expression for a numerically identical solution. However, the paper stops short of providing the curvature expression and how to navigate *p*, *q* for the four different hyperbola configurations. Furthermore, they do not supply a clear explanation or derivation for their expressions.

Goldberg & Sanchez del Rio (2023 ▸) also derive a similar expression for a mirror-centered hyperbola. Nevertheless, the expression requires switching signs depending on the relative values of *p* and *q*, and the slope and curvature are not provided. Additionally, Goldberg & Sanchez del Rio (2023 ▸) only derive the equation for a defocusing hyperbola. The equations here uniquely describe a focusing hyperbola.

## 5. Conclusions

Hyperbolic mirrors are commonly used in optics to focus a converging light source. Such mirrors are often paired with elliptical or parabolic mirrors in a Wolter type configuration for X-ray beamlines. We have provided a novel, closed-form, mirror-centered expression for the shape, slope and curvature of a focusing plane–hyperbolic mirror. The equations depend on optics terms including incident beam angle, and source and image distances. Compared with the canonical hyperbola equation, with eccentricity or semi-axes terms, the expressions herein are far more convenient for optical design. We also observe that each of the four possible hyperbola configurations, focusing or defocusing, convex or concave, are uniquely described by four distinct surfaces. Distinguishing between these cases can be challenging, particularly when analyzing grazing angle mirrors. Table 1 ▸ in Appendix *B*[App appb] concisely summarizes the different configurations. Previous works have relied on power series and polynomial approximations to express similar surfaces, or offer incomplete derivations and explanations. The succinct, exact expression presented here renders metrology, manufacturing and mirror-bending calculations far easier and arbitrarily precise.

## Figures and Tables

**Table 1:** Hyperbola configurations

Purpose	Real focus	Relative source/image distances	Curvature	Equations
Focus	Real image	*p* > *q*	Concave	(14)–(17)
		*p* < *q*	Convex	
Defocus	Real source	*p* > *q*	Convex	(18)–(21)
		*p* < *q*	Concave	

## Appendix A Defocusing the hyperbolic mirror

In a similar form to equations (14) through (17), the complementary equations for a defocusing hyperbolic mirror are as follows,

such that

A defocusing hyperbolic mirror with *q* > *p* produces > 0 and is concave. When *q* < *p*, then  < 0 and the mirror is convex. The real source originates from the hyperbola focus , and the virtual image is focused at .

## Appendix B Summary of hyperbolas

Table 1 ▸ is handy for differentiating between the hyperbola configurations. Note that we only need two sets of equations: one for focusing hyperbolas and one for defocusing hyperbolas.
